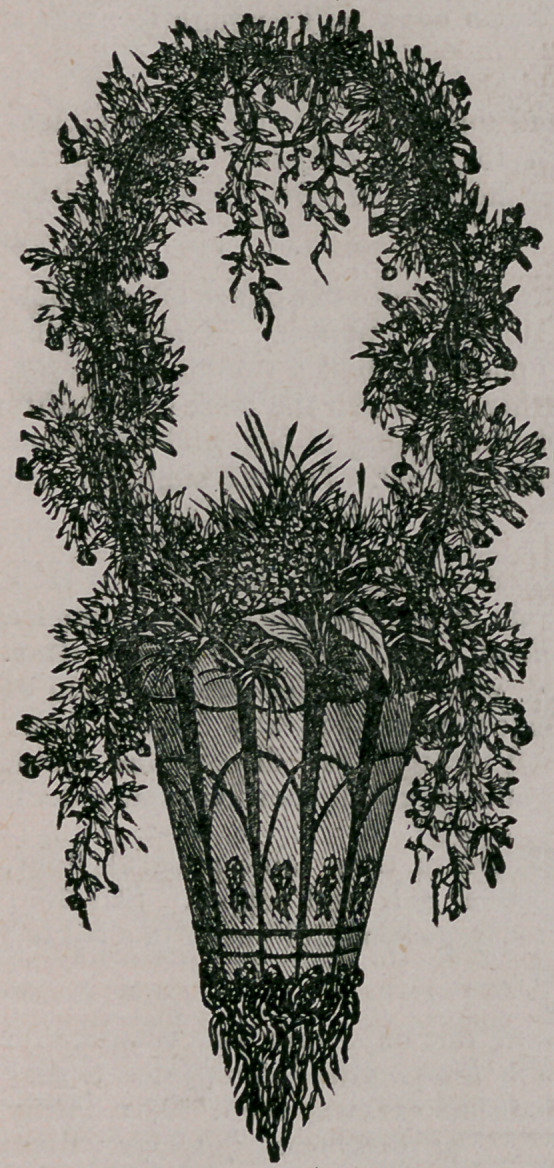# Household

**Published:** 1887-10

**Authors:** 


### HOUSEHOLD.

Rustic Hanging Basket.—The accompanying drawing represents a rustic hanging basket, that any person can make with the common house tools, axe, saw, knife, hammer and a few brads. First, procure from the woods, two or three sticks of iron wood, or such as may suit the fancy. They should be selected, small trees, about three inches in diameter. After selecting the tree, cut it up into pieces fourteen or fifteen inches in length; then, taking one of these round sticks, split off the four sides; this, if it splits well, will give eight pieces from two sticks, the number required to make the basket. The sticks or pieces should be narrower and thinner at one end than the other, as shown in the cut, and rounded at each end. Then procure a block or piece of inch board, and cut out a circular piece about three inches in diameter, slanting it a little so that the pieces will have the taper towards the bottom when tacked to the block. This gives the basket a little flare. They should fit close together at the point where the block is, and may be a little open nearer the top, in order to fill between with moss. Now, the pieces being nailed to the block with brads, begin to ornament it with grape vines and roots. Roots are tacked to the under side of the block, to fill it all up, and at the lower points of the pieces where they match, always keeping in view one thing—to preserve the tapering form and matching the roots in every way that will bring them all towards the centre with uniformity. Next put vines on the sides, as per engraving, bringing two together over the places where the sticks match; also, weave in around the top two vines, in and out alternately, and, fastening with brads, tack roots on the pieces between the ornamental work.

Next put on a handle of grape vine, giving it a single knot; tie at the top to form a loop, interweaving it with a smaller vine; then give the basket a coat of varnish and put in suitable plants. Keep the basket partially in the shade, and occasionally dip it in a barrel of rain water.

Pop Overs.—Two cups of milk, two and one half cups of flour, two eggs butter size of one-half walnut, salt, melt the butter, beat all thoroughly together, put in cups and bake thirty minutes.

Cornmeal Muffins.—One and one-half cups cornmeal, the same of flour, two teaspoonfuls baking powder, half cup sugar, half teaspeonful salt, small teaspoonfnl melted butter, two eggs, milk enough to make a stiff batter.

Tomato Fritters.—One quart stewed tomatoes, one egg, one small teaspoonful soda. Stir in flour enough to make a batter like that for griddle cakes. Have some lard very hot on the stove, drop the batter in a spoonful at a time and fry.

Remedy for Sore Throat.—Buy at a drug store one ounce of camphorated oil, and five cents worth of chlorate of potash. Whenever any soreness appears in the throat, put the potash in half a tumbler of water, and with it gargle the throat thoroughly, then rub the neck thoroughly with the camphorated oil at night before going to bed, and also place around the throat a small strip of woolen flannel. This is a simple, cheap and sure remedy.

Cure for Freckles.—Horseradish grated into a cup of cold sour milk —let it stand twelve hours, then strain and apply two or three times a day—will, it is said, remove freckles from hands or face in a short time. Or, one ounce of lemon juice mixed with a quarter of a drachm of pulverized borax and half a drachm of sugar, will also remove them. Keep the lotion in a glass bottle, corked tightly a few days before using, aud apply to the freckles occasionally, and they will soon be removed.

Egg Omelette.—One pint rich sweet cream, three tablespoonfuls flour, three eggs well beaten, half tablespoonful salt and pepper. Stir flour and milk smooth, add the eggs. Melt a large spoonful butter in a baking pan, pour in, and bake twenty minutes.

Cream Cookies.—One cup sour cream, one cup sugar, one teaspoonful soda and one of cream tartar, with a teaspoonful lemon juice, a very little grated nutmeg and two tablespoonfuls caraway seed. Mix lightly and roll out as soft as possible, using just flour enough to keep them from sticking to the board.

Apple Dumplings.—Sift one quart flour, add half teaspoonful salt, and lard half the size of an egg. Wet up with cold water to a stiff dough. This divide into six or seveu parts. Pare as many good-sized apples, cut through the middle, removing the core; cover with the dough, pressing the edges together till no seam remains; then when all are ready, roll two or three times over in dry flour, and drop into boiling water. Boil steadily half an hour, not once lifting the lid till ready to remove to the table. Eat with cream and sugar sprinkled with grated nutmeg.

## Figures and Tables

**Figure f1:**